# Isolated iliac cryptococcosis in an immunocompetent patient

**DOI:** 10.1371/journal.pntd.0006206

**Published:** 2018-03-29

**Authors:** Junjun Sang, Yali Yang, Yibin Fan, Guizhen Wang, Jiu Yi, Wei Fang, Weihua Pan, Jinhua Xu, Wanqing Liao

**Affiliations:** 1 Shanghai Key Laboratory of Molecular Medical Mycology, Department of Dermatology and Venereology, Changzheng Hospital, Shanghai, China; 2 Department of dermatology, Fuzhou General Hospital, Fuzhou, China; 3 Department of dermatology, Zhejiang Provincial People’s Hospital, Hangzhou, Zhejiang, China; 4 Emergency room, Shanghai Tenth People’s Hospital of Tongji University, Shanghai, China; 5 Department of Dermatology, Huashan Hospital, Fudan University, Shanghai, China; Universidad de Antioquia, COLOMBIA

## Introduction

### Overview

Cryptococcal osteomyelitis is an infrequent infection that is usually associated with disseminated cryptococcosis or underlying immunocompromised conditions. Here we describe a rare case of isolated iliac cryptococcosis in an immunocompetent patient. Through histological, microbial, and molecular biological examinations, the pathogen was finally identified as *Cryptococcus neoformans* VNI genotype, which likely originated from environmental bird droppings. The clinical isolate was hypomelanized but fully virulent in a mouse infection model. The patient displayed a lower CD4^+^ T-lymphocyte ratio, reduced serum interferon gamma (IFN-γ) and interleukin 12 (IL-12) levels, and a dysregulated transcriptional profile of blood leukocytes compared with the healthy host. After surgical excision and 34 weeks of antifungal treatment, the patient achieved clinical cure. Our study suggests that cryptococcosis development is closely associated with the interaction of the fungal agent and host immunity. Accurate diagnosis of bone cryptococcosis depends mainly on histological and fungal examinations. A combination of an antifungal agent treatment regimen and surgery are highly effective for resolving bone cryptococcosis.The *C*. *neoformans* species complex primarily causes opportunistic infections in immunocompromised patients, commonly involving the lungs and central nervous system [1]. Skeletal involvement is infrequent and usually associated with disseminated cryptococcosis or underlying predisposing conditions such as sarcoidosis, tuberculosis, or other systemic disorders [2,3]. We present here a rare case of isolated skeletal cryptococcosis in an immunocompetent host and further discuss the manifestation of the infection from both pathogen and host perspectives by in vitro and in vivo assays. Informed written consent was obtained prior to publication of the case details.

### Case report

A previously healthy 46-year-old woman presented with left hip pain of undetermined origin for 2 months. The pain was not alleviated after symptomatic treatment. Physical examination showed slight swelling of the left hip with no broken skin, and the patient presented a limp. There was no obvious spine deformity or muscle atrophy nor tenderness or a mass detected upon palpation. The Patrick sign and ankle or knee jerk reflexes were normal. The patient had no fever, cough, coma, headaches, or weight loss.

**Fig 1:**
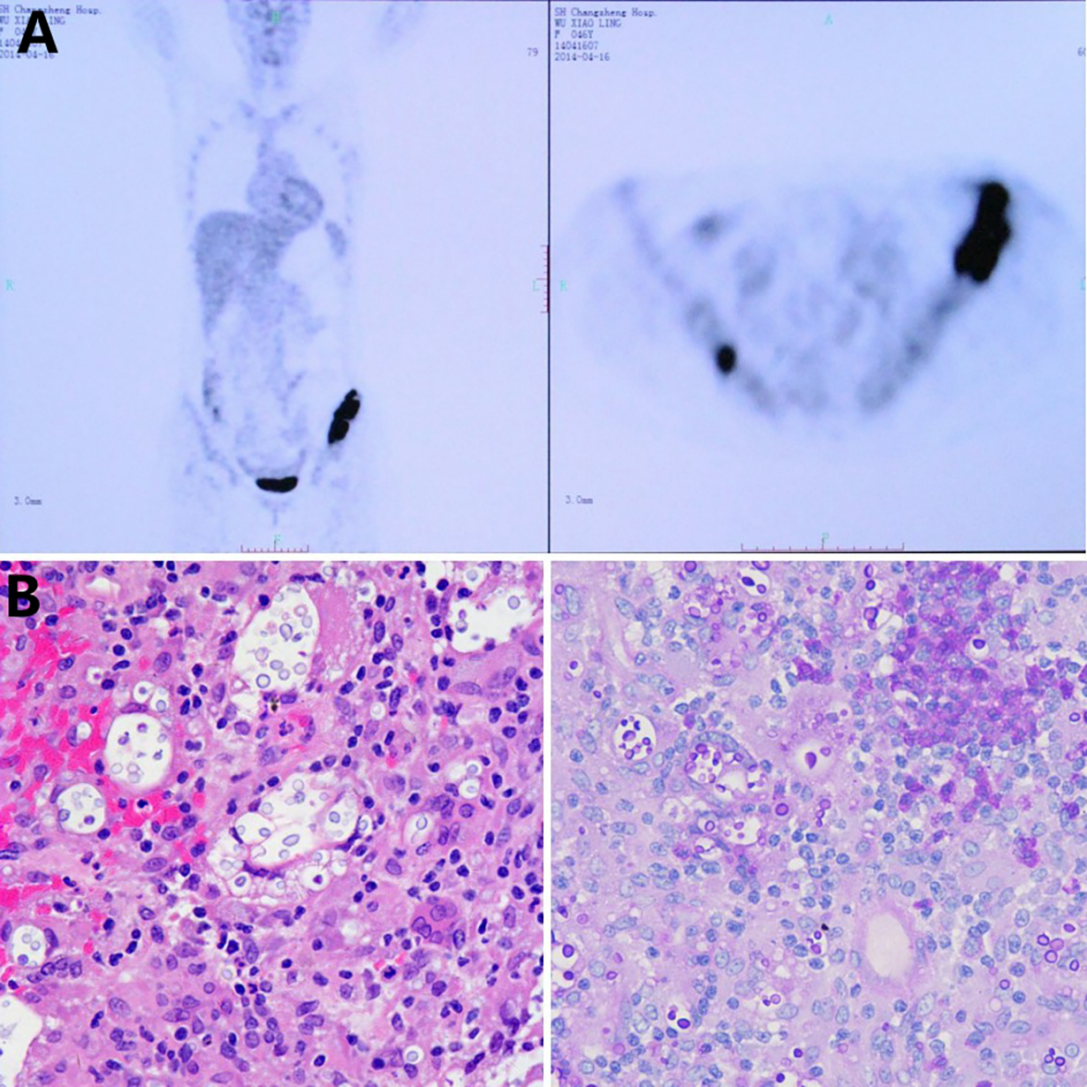
^18^FDG-PET/CT scan and histological examination. **(A)** ^18^FDG-PET/CT indicated hypermetabolic lesions in the bilateral iliac crest, especially in the left iliac crest. **(B)** Histological examinations (HE stain, left panel; PAS stain, right panel) after left iliac biopsy revealed round yeast cells. HE, hematoxylin and eosin; PAS, periodic acid–Schiff; ^18^FDG-PET/CT, ^18^fluorodeoxyglucose positron emission tomography and computed tomography.

Most of the laboratory examinations were normal, such as blood biochemistry tests, HIV test, antinuclear antibodies, and tumor markers. The blood profile revealed 79.8% neutrophils (high) and 12.9% lymphocytes (low). CD4 and CD3 counts were further performed, and the results were negative (CD3: 63.6%, normal; CD3 absolute value: 284; CD4: 25.1%, low; CD4 absolute value: 112; CD8: 37.5%, high; CD8 absolute value: 168; CD4/CD8: 0.67%, low). ^18^fluorodeoxyglucose positron emission tomography and computed tomography (^18^FDG-PET/CT) indicated hypermetabolic lesions in the bilateral iliac crest, especially in the left iliac crest (Fig 1A). The pulmonary computed tomography (CT) scan demonstrated multiple obsolete bilateral lung lesions (S1 Fig). The patient underwent a left iliac bone biopsy. Microscopic examination and fungal culture displayed abundant small round organisms, which were suspected to be *C*. *neoformans* based on morphological characteristics and histopathological findings (Fig 1B). The blood cryptococcal antigen test was positive (>1:5,120). The cerebrospinal fluid (CSF) biochemistry and microbial tests were negative.

After excision of skeletal cryptococcosis, treatment with liposomal amphotericin B (4 mg/kg/day) and flucytosine (100 mg/kg/day) was administered for 2 weeks; voriconazole (0.4 g/day) was then administered for 8 weeks. The patient was discharged to her home on oral fluconazole (800 mg/day for 12 weeks) for consolidation, which was then tapered to 400 mg/day for 12 weeks. The serum cryptococcal antigen titers were 1:2,560 after 1 month of treatment, 1:1,280 after 2 months of treatment, and 1:2 at 1 year postdischarge. The pelvic CT findings at 1 year postdischarge suggested clinical cure (S1 Fig).

**Fig 2:**
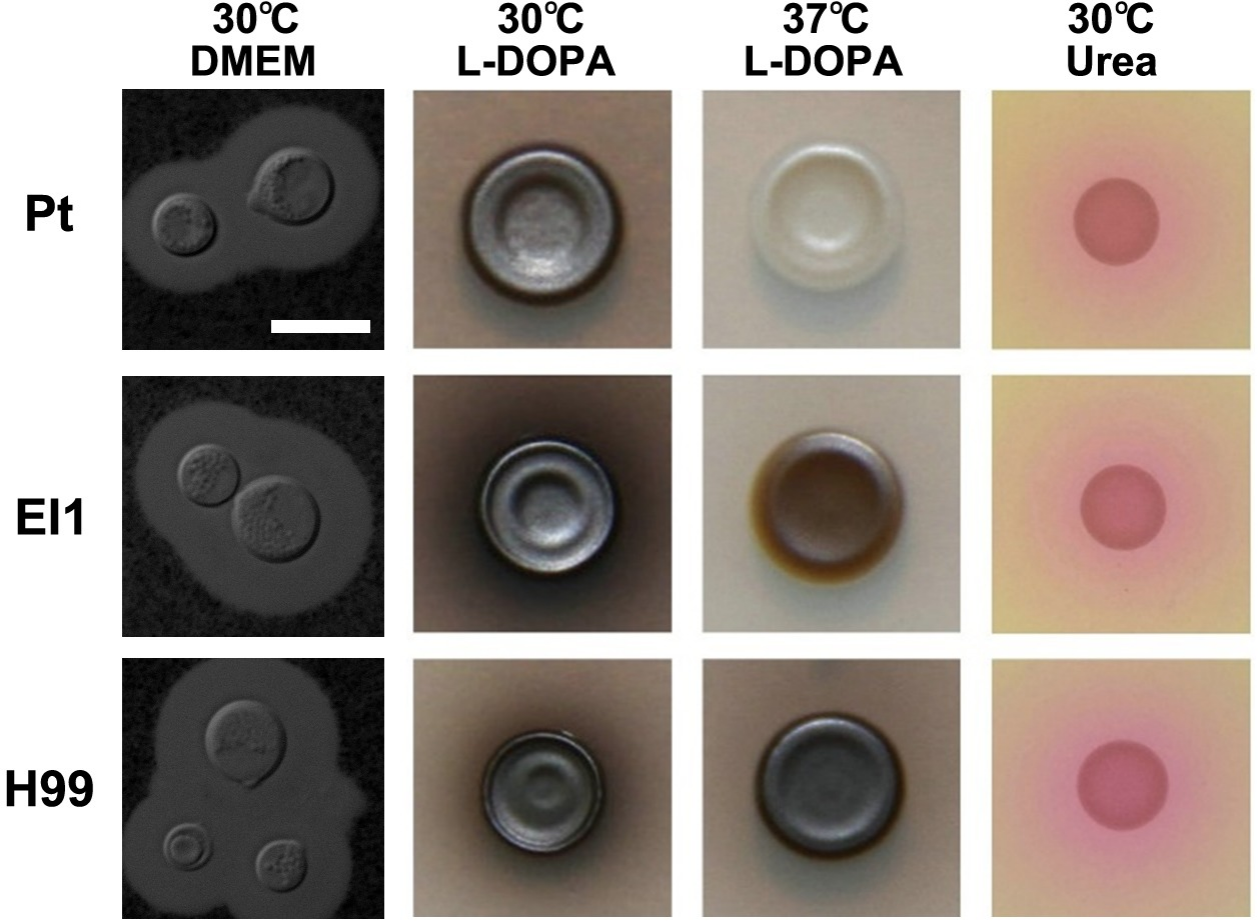
In vitro assay of virulence factors. Different media (DMEM liquid, L-DOPA, and Christiansen’s urea agars) were utilized to test the production of major virulence factors (such as capsule, melanin, and urease) in *C*. *neoformans*. Compared with the hypervirulent strain (H99) or environmental isolate (El1), the clinical isolate (Pt) displayed a significant defect in melanization at host temperature but not in capsule or urease production.

**Fig 3:**
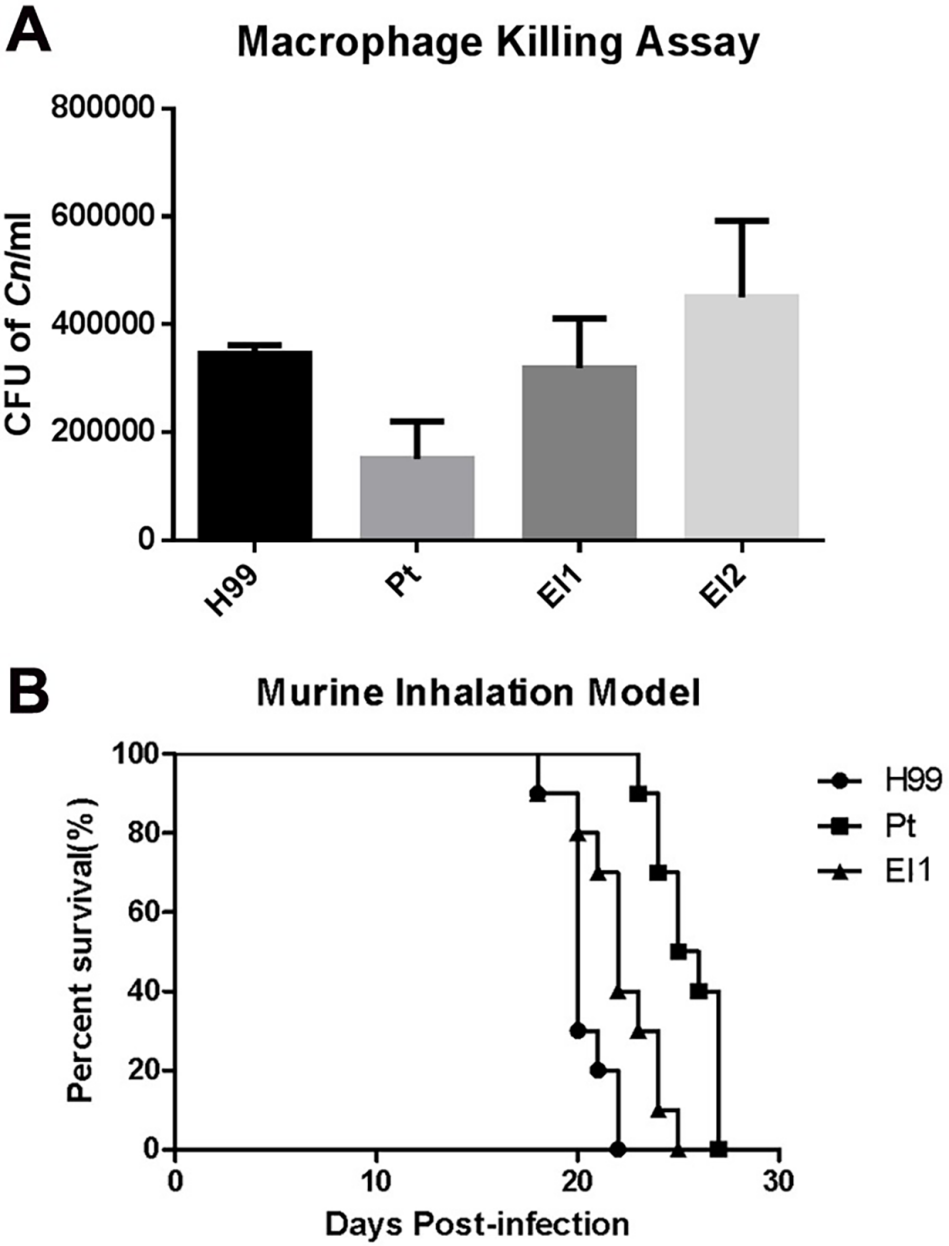
Macrophage killing assay and murine inhalation model. Both macrophage **(A)** (*P* < 0.01) and animal **(B)** (*P* < 0.001) infection experiments displayed attenuated virulence of the clinical isolate compared with the hypervirulent strain H99.

We examined her epidemiological history to determine the potential source of exposure to cryptococcosis. Three yeast colonies were finally isolated from 20 samples of wild bird droppings near the patient’s residence. The internal transcribed spacer (ITS) sequencing [4] and PCR fingerprinting [5] confirmed that all the clinical and environmental isolates were *C*. *neoformans* VNI genotype (S2 Fig and S1 Table). In vitro phenotypic assays showed that the clinical isolate exhibited significant hypomelanization at 37°C but normal production of capsule or urease (Fig 2). Furthermore, the clinical isolate was fully virulent in vivo, although it displayed extended survival in a mouse inhalation model (*P* < 0.001) (Fig 3B) and exhibited a significant reduction in intracellular survival compared with the standard strain (H99) or the environmental isolate (El1) (*P* < 0.01) (Fig 3A).

Cytokine and transcriptional profiles were examined in patient serum samples by ELISA and RNA-seq analysis, respectively. The results revealed a significant reduction of interferon gamma (IFN-γ) (*P* < 0.001) and interleukin 12 (IL-12) (*P* < 0.05) compared with the healthy control groups (S3 Fig). The transcriptional profiles (Gene Expression Omnibus Accession number: GSE108534) showed an enrichment of a primary immunodeficiency phenotype, such as T cell signaling pathways, the hematopoietic cell lineage, the nuclear factor kappa-light-chain-enhancer of activated B cells (NF-ĸB) signaling pathway, antigen processing and presentation, and natural killer cell-mediated cytotoxicity (S3 Fig). All the materials and methods are provided in S1 Text.

## Discussion

Skeletal cryptococcosis is usually secondary to direct inoculation during trauma or hematogenous dissemination [3]. Since the patient had multiple obsolete pulmonary lesions and a high blood cryptococcal antigen titer but no history of trauma, we speculated that the agent originated from primary pulmonary lesions. PCR fingerprinting revealed that all isolates grouped into the VNI genotype [6]. Thus, an environmental carrier (bird droppings) of *C*. *neoformans* might be the ultimate source of skeletal infection in this case.

Pathogenesis and progression of cryptococcosis depends on the interaction between the fungal agent and host immunity. The clinical agent in this study showed complete virulence in vivo and ex vivo, although its virulence was inferior to the standard strain (H99) or environmental isolates, which might be partially attributed to its hypomelanization at host temperature. Several laboratory test indices, such as HIV, autoimmune antibodies, and tumor markers, were normal, and the patient’s medical history showed no medical use of steroids or immunosuppressants. The patient displayed a lower CD4^+^ T-lymphocyte ratio, significantly reduced serum Th1 cytokine (IFN-γ and IL-12) responses, and a dysregulated transcriptional profile of blood leukocytes, as compared with healthy populations. Two potential scenarios may explain this phenomenon. On the one hand, the presence of cryptococcal antigens (such as glucuronoxylomannan) or previously applied anti-inflammatory therapy might cause a slight compromise of host cellular immunity [7,8]. On the other hand, the abnormalities in immunological indices might originate from host subclinical immune impairment, which remains to be further confirmed.

We reviewed the literature on immunological analyses in apparently normal individuals with cryptococcosis. The *IL12RB1* gene mutation has been reported in pediatric patients with cryptococcal osteomyelitis or disseminated cryptococcosis [9,10]. Several studies have also suggested that genetic polymorphisms of some immune molecules (such as mannose-binding lectin, dectin-2, and Fcγ receptor IIB) are closely associated with cryptococcosis in immunocompetent populations [11,12,13]. All these factors were associated with host immune functions essential for fungal control and containment, such as phagocytic activity and T cell responses [14,15]. Thus, subclinical immune impairment might be a non-negligible factor in apparently healthy patients with cryptococcosis.

The clinical symptoms and radiological findings of skeletal cryptococcosis were nonspecific. A correct diagnosis still relied on histopathological and fungal examinations of lesion specimens. After focal cleaning operations, multiple antifungal agents were sequentially administered, such as liposomal amphotericin B, flucytosine, and azole agents, and the patient achieved clinical cure.

## Conclusions

Isolated skeletal cryptococcosis is rare but may occur in healthy individuals after daily exposure to the organism. Herein, the clinical isolate likely originated from environmental bird droppings disseminated from the pulmonary lesion to the hip. The development of cryptococcosis was closely associated with the interaction of the fungal agent and host immunity. Accurate diagnosis of bone cryptococcosis depends mainly on histological and fungal examinations. A combination antifungal agent treatment regimen and surgery were quite effective for resolving bone cryptococcosis.

## Ethics statement

This study was approved by the Committee on Ethics of Biomedicine Research, Changzheng Hospital. All subjects gave written informed consent in accordance with the Declaration of Helsinki. The animal experiments were carried out in strict accordance with the recommendations in the Regulations for the Administration of Affairs concerning Experimental Animals of the State Science and Technology Commission (China). The protocol was approved by the Animal Experiment Center of Second Military Medical University.

## Supporting information

S1 Text Materials and methods. (DOCX)

S1 Fig CT scans. **(A)** The pulmonary CT scan on admission showed multiple obsolete lesions in the bilateral upper lobes and lower lobe of the left lung. **(B)** The pelvic CT scan at 1 year post-discharge suggested the infectious lesions had disappeared. CT, computed tomography.
(TIF)

S2 Fig Molecular-type identification of clinical and environmental isolates by PCR fingerprinting. All isolates were identified as VNI genotype. Pt is the cryptococcal strain isolated from the iliac lesion of the patient. EI denotes environmental isolates from the patient’s area of residence.
(TIF)

S3 Fig Serum cytokine and transcriptional profile analyses. **(A)** The serum cytokine assay revealed a significant reduction of IFN-γ and IL-12 compared with the control groups. *** means *P* < 0.001. * means *P <* 0.05. **(B)** Bioinformatics analysis of RNA sequencing in the patient serum sample displayed an enrichment of several immunological pathways. IFN-γ, interferon gamma; IL-12, interleukin 12.
(TIF)

S1 Table Strains used in this study. Pt, cryptococcal strain isolated from the biopsy specimen; EI-1,2,3, cryptococcal strains isolated from bird droppings near the patient’s residence. The other strains were provided by our laboratory.
(DOCX)
