# Supplementary material for: Isolated iliac cryptococcosis in an immunocompetent patient
Source: PLoS Negl Trop Dis. 2018 Mar 29;12(3):e0006206. doi: 10.1371/journal.pntd.0006206 (PMC5875738; doi:10.1371/journal.pntd.0006206)
Supplement: S1 Text — (DOCX) [file pntd.0006206.s001.docx]

## Supplemental data

## S1Text. Materials and Methods

### Strains and Media

The *C. neoformans* strains used in this study were stored at 4°C and cultured at 30°C on yeast peptone dextrose (YPD) agar medium. Stress media were created by adding different stress-inducing agents into yeast nitrogen base (YNB) agar medium or YPD agar medium before autoclaving.

### Genotype and Serotype Analysis

Genomic DNA was extracted according to the procedures described by Tamura et al.[4], and the internal transcribed spacer (ITS) regions including the 5.8S rDNA clusters were amplified using the ITS4 and ITS5 sets (ITS4:5-TCCTCCGCTTATTGATATGC-3, ITS5: 5-GGAAGTAAAAGTCGTAACAAGG-3). Acquired sequences were submitted to GenBank and BLAST analysis to determine the identity of the isolates. PCR fingerprinting using the single primer M13 (5-GAGGGTGGCGGTTCT-3) was used to identify the genotype and serotype of the *C. neoformans* isolates [5].

### *In vitro* Phenotypic Assays

Yeast cells were counted using a hemocytometer, and the cell density was adjusted to 10^6^ cells/ml and 10^3^ cells/ml in YPD medium. For the growth assays, the cells were grown in a 96-well plate. Six wells were assessed for each strain. The growth rate was measured at 600 nm at 6-hour intervals using an OPTIMA microplate spectrophotometer. For *in vitro* stress assays, each strain was incubated to saturation at 30°C in YPD medium, washed, diluted to 2.5×10^8^ cells/ml in phosphate-buffered saline (PBS), and spotted (4 µl) onto YNB or YPD agar medium containing different stress-inducing agents. For the oxidative and nitric oxide (NO) stress test, 2 mM H_2_O_2_ or 0.75 mM NaNO_2_ was added to the YNB agar medium. For the osmotic stress and high-salt sensitivity test, we added 1.5 M sorbitol, 1.5 M NaCl or 1.5 M KCl to the YPD agar medium. To examine the cell wall integrity, the cells were spotted onto YPD agar medium containing 0.02% SDS, 0.1% caffeine, or 0.5% Congo Red. The spotted cells were then incubated at 30°C for 3 days and photographed. To evaluate temperature stress, the YPD plates were incubated at 30°C, 37°C, and 39°C.

For capsule induction, the yeast cells were cultured in liquid Sabouraud medium overnight, washed with sterile PBS, and cultured in dilute Sabouraud (Difco^TM^ 238230) 1/10 in 50 mM MOPS (Sigma, Cat. No M1442-L), pH 7.3 at a cell density of 5×10^6^ cells/ml. The cultures were incubated at 37°C for 24 hours. For the melanization test, cells were spotted onto L-DOPA agar and incubated for 3 days at 30°C and 37°C. The urea plate was used to detect urease activity in the WT and mutant yeast strains.

### Macrophage and Murine Infection Experiments

We assessed the clinical and environment strains for survival within macrophages. Yeast strains were incubated overnight at 30°C. J774A.1 macrophages at a concentration of 10^6^ cells/well were activated with interferon-γ (INF-γ) and lipopolysaccharide for 18 hours. The activated macrophages were co-incubated with 10^6^ yeast cells for 2 hours at 37°C in 5% CO_2_ to allow phagocytosis. Prior to co-incubation, the yeast cells were incubated with the monoclonal antibody mAb18B7 for 1 hour. Extracellular yeast were then removed by three washes with PBS, and the washed monolayer of macrophages containing yeast were co-incubated for 24 hours. The macrophages were then lysed with 0.5% SDS, and the lysates were diluted and plated on YPD to count the number of viable yeast cells present following incubation at 30°C for 3 days.

BALB/c female mice were used in this study. Mice were obtained from the Institute of Zoology, Chinese Academy of Sciences, and raised under specific pathogen–free conditions in cages covered with a filter top. The strains studied herein were grown for 24 hours in YPD and washed three times with PBS. The concentration of the original culture was diluted in PBS to a final concentration of 2×10^6^ cells/ml, followed by the delivery of 25 µl (50,000 cells) intranasally by dropping the inoculum into the nares. The survival rates were determined for groups of ten animals per strain. The animals were sacrificed when a loss of 20% body weight was observed from the peak body weight. Other signs of sickness were monitored, including lethargy and lack of activity and grooming (indicated by ruffled fur).

### Serum Sample Processing and Cytokine Immunoassays

Two batches of patient serum samples (10 ml) were obtained prior to treatment. Serum samples in the two control groups were isolated from 8 healthy volunteers (4 females, 4 males, 32-50 years old) who were randomly divided into two groups. Serum was obtained by low-speed centrifugation, repacked, and stored at -80°C. The enzyme-linked immunosorbent assay (ELISA) was used to determine the serum concentrations of INF-γ, tumor necrosis factor-α (TNF-α), interleukin-2 (IL-2), IL-13, IL-4, IL-12, IL-22, IL-17A, and granulocyte-macrophage colony-stimulating factor (GM-CSF). The ELISA kits were obtained from ProImmune Ltd (Oxford, OX, UK).

### RNA-seq Assay

Total RNA was extracted using the PAXgene Blood RNA Kit (Cat#762174, Qiagen) following the manufacturer’s instructions, and the RIN number was determined to evaluate the RNA integrity using an Agilent Bioanalyzer 2100 (Agilent technologies, Santa Clara, CA, US). Qualified total RNA was further purified using the RNAClean XP Kit (Cat A63987, Beckman Coulter, Inc. Kraemer Boulevard Brea, CA, USA) and RNase-Free DNase Set (Cat#79254, QIAGEN, GmBH, Germany).

Total RNA was isolated using the PAXgene Blood RNA Kit (Qiagen, Germany). Strand-specific libraries were prepared using the TruSeq® Stranded Total RNA Sample Preparation kit (Illumina, USA) following the manufacturer’s instructions. Briefly, The poly-A containing mRNA molecules were purified using poly-T oligo-attached magnetic beads. Following purification, the mRNA was fragmented into small pieces using divalent cations at 94℃ for 8 min. The cleaved RNA fragments are copied into first-strand cDNA using reverse transcriptase and random primers, followed by second-strand cDNA synthesis using DNA Polymerase I and RNase H. These cDNA fragments were then subjected to an end repair process, the addition of a single ‘A’ base, and then ligation of the adapters. The products are then purified and enriched by PCR to create the final cDNA library. Purified libraries were quantified using a Qubit® 2.0 Fluorometer (Life Technologies, USA) and validated with an Agilent 2100 bioanalyzer (Agilent Technologies, USA) to confirm the insert size and calculate the mole concentration. The cluster was generated by cBot with the library diluted to 10 pM and then sequenced on the Illumina HiSeq 2500 (Illumina, USA). Library construction and sequencing were performed by Shanghai Biotechnology Corporation.

### Statistical Analyses

The data are representative of at least three independent experiments. Values represent the means and standard deviations (SD) of triplicates. Survival analyses were conducted using Kaplan-Meier survival curve methodology with the log-rank (Mantel-Cox) test. The virulence data were analyzed with the log-rank test for statistical differences. A two-tailed unpaired Student’s t test was used for all other statistical tests between two groups. All statistical analyses were performed using the GraphPad Prism 5.0 software program. *P*≤0.05 was considered statistically significant.

Supplemental Figures.

S1Figure. CT scans. A. the pulmonary CT scan on admission showed multiple obsolete lesions in the bilateral upper lobes and lower lobe of the left lung. B. The pelvic CT scan at 1 year post-discharge suggested the infectious lesions had disappeared.

S2Figure. Molecular type identification of clinical and environmental isolates by PCR fingerprinting. All isolates were identified as VNI genotype. Pt, cryptococcal strain isolated from the iliac lesion of the patient. EI denotes environmental isolates from the patient’s area of residence.

S3Figure. Serum cytokine and transcriptional profile analyses. A. The serum cytokine assay revealed a significant reduction of IFN-γ and IL-12 compared with the control groups. *** means *P*＜0.001. * means *P*＜0.05. B. Bioinformatics analysis of RNA sequencing in the patient serum sample displayed an enrichment of several immunological pathways.

S1Table. Strains used in this study. Pt, cryptococcal strain isolated from the biopsy specimen; EI-1,2,3, cryptococcal strains isolated from bird droppings near the patient’s residence. The other strains were provided by our laboratory.

| No. | Strains | Molecular type | Accession No. in GenBank |
| --- | --- | --- | --- |
| 1 | Pt | *C. neoformans* VNI | SUB3215561 |
| 2 | EI-1 | *C. neoformans* VNI | SUB3215565 |
| 3 | EI-2 | *C. neoformans* VNI | SUB3215569 |
| 4 | EI-3 | *C. neoformans* VNI | SUB3215571 |
| 5 | H99 | *C. neoformans* VNI |  |
| 6 | WM148 | *C. neoformans* VNI |  |
| 7 | WM626 | *C. neoformans* VNII |  |
| 8 | WM628 | *C. neoformans* VNIII |  |
| 9 | WM629 | *C. neoformans* VNIV |  |
| 10 | WM179 | *C. gattii* VGI |  |
| 11 | WM178 | *C. gattii* VGII |  |
| 12 | WM161 | *C. gattii* VGIII |  |
| 13 | WM779 | *C. gattii* VGIV |  |
