# Supplementary figures and images for: Isolated iliac cryptococcosis in an immunocompetent patient
Source: PLoS Negl Trop Dis. 2018 Mar 29;12(3):e0006206. doi: 10.1371/journal.pntd.0006206 (PMC5875738; doi:10.1371/journal.pntd.0006206)

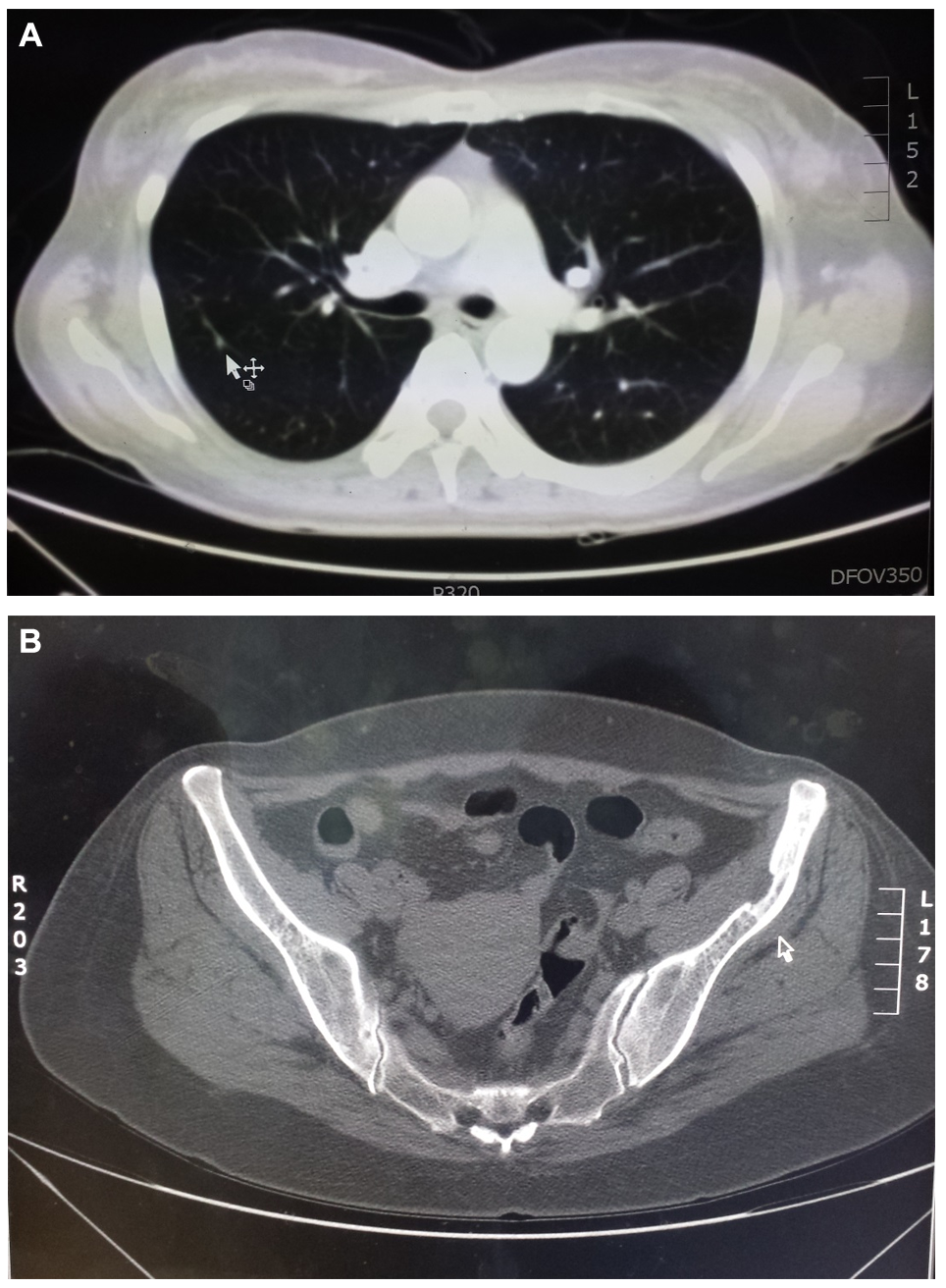

Supplement: S1 Fig — (A) The pulmonary CT scan on admission showed multiple obsolete lesions in the bilateral upper lobes and lower lobe of the left lung. (B) The pelvic CT scan at 1 year post-discharge suggested the infectious lesions had disappeared. CT, computed tomography. (TIF) [file pntd.0006206.s002.tif]

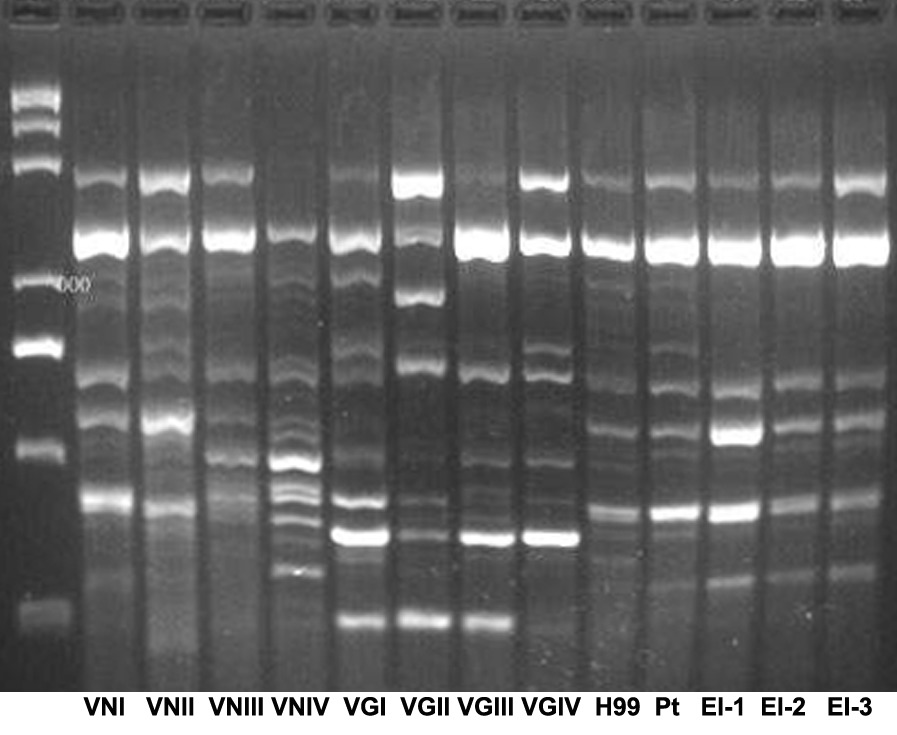

Supplement: S2 Fig — All isolates were identified as VNI genotype. Pt is the cryptococcal strain isolated from the iliac lesion of the patient. EI denotes environmental isolates from the patient’s area of residence. (TIF) [file pntd.0006206.s003.tif]

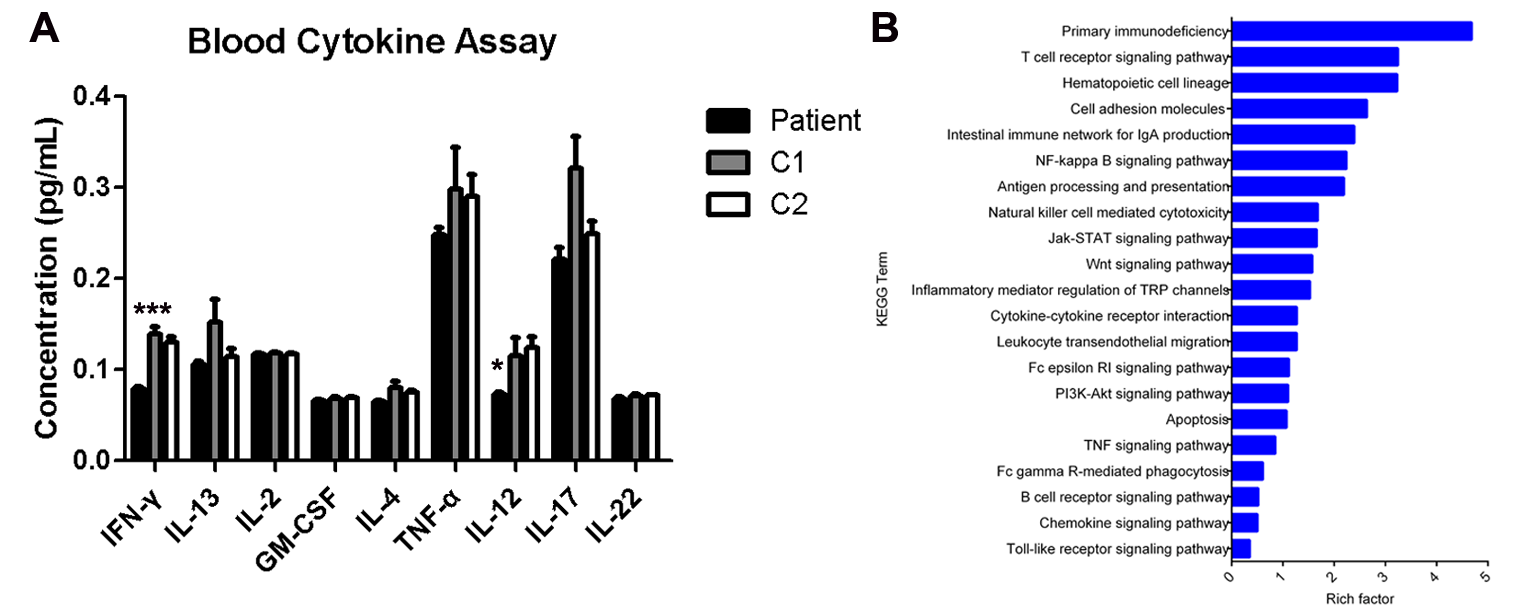

Supplement: S3 Fig — (A) The serum cytokine assay revealed a significant reduction of IFN-γ and IL-12 compared with the control groups. *** means P < 0.001. * means P < 0.05. (B) Bioinformatics analysis of RNA sequencing in the patient serum sample displayed an enrichment of several immunological pathways. IFN-γ, interferon gamma; IL-12, interleukin 12. (TIF) [file pntd.0006206.s004.tif]
