# Supplementary material for: Isolated iliac cryptococcosis in an immunocompetent patient
Source: PLoS Negl Trop Dis. 2018 Mar 29;12(3):e0006206. doi: 10.1371/journal.pntd.0006206 (PMC5875738; doi:10.1371/journal.pntd.0006206)
Supplement: S1 Table — Pt, cryptococcal strain isolated from the biopsy specimen; EI-1,2,3, cryptococcal strains isolated from bird droppings near the patient’s residence. The other strains were provided by our laboratory. (DOCX) [file pntd.0006206.s005.docx]

S1 Table. Strains used in this study. Pt, cryptococcal strain isolated from the biopsy specimen; EI-1,2,3, cryptococcal strains isolated from bird droppings near the patient’s residence. The other strains were provided by our laboratory.

| No. | Strains | Molecular type | Accession No. in GenBank |
| --- | --- | --- | --- |
| 1 | Pt | *C. neoformans* VNI | SUB3215561 |
| 2 | EI-1 | *C. neoformans* VNI | SUB3215565 |
| 3 | EI-2 | *C. neoformans* VNI | SUB3215569 |
| 4 | EI-3 | *C. neoformans* VNI | SUB3215571 |
| 5 | H99 | *C. neoformans* VNI |  |
| 6 | WM148 | *C. neoformans* VNI |  |
| 7 | WM626 | *C. neoformans* VNII |  |
| 8 | WM628 | *C. neoformans* VNIII |  |
| 9 | WM629 | *C. neoformans* VNIV |  |
| 10 | WM179 | *C. gattii* VGI |  |
| 11 | WM178 | *C. gattii* VGII |  |
| 12 | WM161 | *C. gattii* VGIII |  |
| 13 | WM779 | *C. gattii* VGIV |  |
